# Dr. Charles Alfred Greene

**Published:** 1911-03

**Authors:** 


Dr. Charles Alfred Greene, died at his home, Windom, Minn., November 10, 1910, from heart disease, aged 65 years. He was a graduate of the University of Buffalo, class of 1873.

